# Editorial on Special Issue “Plasmid DNA for Gene Therapy and DNA Vaccine Applications”

**DOI:** 10.3390/pharmaceutics17050630

**Published:** 2025-05-09

**Authors:** Urška Kamenšek

**Affiliations:** 1 Department of Experimental Oncology, Institute of Oncology Ljubljana, SI-1000 Ljubljana, Slovenia; ukamensek@onko-i.si; 2 Biotechnical Faculty, University of Ljubljana, SI-1000 Ljubljana, Slovenia

## 1. Introduction

While the use of plasmid DNA for therapeutic delivery is not a new concept, the field has witnessed remarkable innovation in recent years, ranging from novel plasmid designs to improvements in delivery platforms and clinical positioning of DNA-based therapies. Plasmid DNA technologies include applications ranging from the expression of therapeutic proteins and vaccine antigens to genome editing components and viral vector elements. Thanks to advances in artificial gene synthesis and molecular engineering, plasmids and other DNA vectors can now be more easily designed and optimized for targeted and high-level expression. On the other hand, innovations in plasmid backbone design and purification techniques have enabled the production of safer and more efficient constructs for clinical use.

## 2. Overview of the Published Articles

The Special Issue “Plasmid DNA for Gene Therapy and DNA Vaccine Applications” showcases 25 peer-reviewed studies, highlighting the maturity and future potential of plasmid DNA in therapeutic gene delivery and vaccination. The two editions of this Special Issue provide valuable insights into vector design, delivery methods, underlying mechanisms and therapeutic applications of plasmid DNA, from DNA vaccination to gene therapy for cancer and other diseases.

### 2.1. Optimization of DNA Vectors

The fundamental step for an efficient application of plasmid DNA lies in the optimization of the vector itself. Several contributions in this Special Issue deal with this crucial step. One study evaluated different promoters for plasmid DNA ternary complexes and identified the chicken beta actin hybrid promoter as particularly effective for DNA vaccination (Contribution 1). Another work demonstrated that transcription factor binding sites critically influence the expression pattern and persistence of transgene expression in vivo (Contribution 2).

A review of the use of DNA replicons derived from alphavirus and flavivirus genomes illustrated their ability to enhance expression via RNA amplification, even at lower doses, showing promise in cancer and infectious disease models (Contribution 3). Synthetic PCR-generated transcriptionally active fragments were also evaluated as substitutes for conventional plasmids, offering streamlined approaches for applications such as live virus vaccine production (Contribution 4). While these simplified vectors are suitable for ex vivo applications, their in vivo use raises concerns due to the risks of insertional mutagenesis associated with their linear nature.

### 2.2. Optimization of Plasmid DNA Delivery Methods

Physical and chemical delivery methods continue to define the success of plasmid DNA-based therapies; thus, delivery optimization was another dominant theme in this Special Issue. Electroporation remains the most established method for in vivo plasmid delivery, and several studies addressed its refinement. A comparative study in 2D and 3D cancer cell models demonstrated the importance of pulse protocols for maximizing gene delivery efficiency (Contribution 5). Nanosecond electric fields demonstrated superior transfection efficiencies compared to microsecond pulses in various cell types (Contribution 6). A new device for gene electrotransfer to skin cells was developed, offering improved efficiency through an optimized electrode design and pulse protocols (Contribution 7). Commercial porous cell culture inserts were repurposed for gene electrotransfer, achieving high transfection efficiencies with low voltage pulses, bypassing the use of expensive electroporators (Contribution 8). Efficiency of in vivo electroporation was once again demonstrated to be enhanced by pretreatment with hyaluronidase, which improves plasmid distribution within tissues (Contribution 9), while sucrose was shown to enhance electrotransfer via activation of phospholipase A2 (Contribution 10).

Beyond electroporation, lipid-based systems and milk-derived exosomes were studied as biocompatible carriers with the potential to improve systemic delivery and targeting. Lipid nanoparticles generated via a microfluidic platform were optimized for efficient DNA delivery, achieving high transfection efficiencies with low cytotoxicity (Contribution 11), while bovine milk-derived exosomes were proposed as a scalable, natural delivery system for plasmid DNA, overcoming many of the limitations of conventional vectors (Contribution 12). A study comparing thin-film rehydration and microfluidic-based production of DNA-liposome complexes showed how nanostructural differences in formulation affect immunogenicity and potency (Contribution 13). These advances collectively highlight the diversity of physical, chemical, and biological methods now available for optimizing plasmid delivery, supporting their broader clinical translation.

### 2.3. Mechanistic Insights into Plasmid DNA Delivery

Several papers focused on mechanistic insights into the delivery and expression of plasmid DNA, which are vital to rationally improving gene therapy protocols. The fate of antibody-encoding plasmid DNA after intramuscular electroporation confirmed the importance of nuclear retention for sustained expression (Contribution 14). The acute changes in gene expression after intratumoral DNA electrotransfer were characterized, identifying key pathways that are modulated in response to DNA or to pulse application (Contribution 15). It was shown that pulsed electric fields can induce STING activation independent of the presence of DNA, confirming important immunomodulatory effects of electroporation itself (Contribution 16). A systematic review and meta-analysis identified key electrical and biological parameters influencing gene electrotransfer efficiency, providing valuable guidelines for future optimization efforts (Contribution 17).

### 2.4. DNA Vaccination: Applications Against Infectious Disease

DNA vaccination remains one of the most promising applications of plasmid technology, offering adaptability and speed in response to emerging infectious diseases. The feasibility of gene electrotransfer for vaccination was demonstrated on a model of the COVID-19 vaccine, highlighting the importance of target tissue and antigen selection for the desired effects (Contribution 18). Another paper reported on a DNA prime/protein boost strategy against Campylobacter jejuni in poultry, achieving modest immune responses and emphasizing the need for further optimization (Contribution 19). These studies demonstrate the continuing evolution of plasmid DNA vaccines and their potential in both human and veterinary medicine.

### 2.5. Cancer Gene Therapy and DNA Cancer Vaccines

Cancer-related applications were a major focus in both editions, with plasmid-based strategies applied to immunotherapy and combination regimens. Combining IL-12 gene electrotransfer with anti-PD-1 checkpoint inhibition enhanced tumor infiltration by T cells and improved therapeutic outcomes in metastatic melanoma models (Contribution 20). A DNA vaccine combined with immune checkpoint blockade improved the immune responses in an orthotopic glioblastoma model (Contribution 21). Mesenchymal stem cells engineered via gene electrotransfer to express IL-12 significantly delayed tumor progression in melanoma models, demonstrating a safe and effective anti-tumor strategy (Contribution 22). A review summarized the therapeutic applications of plasmid DNA in cancer, highlighting strategies that stimulate immune responses and directly kill tumor cells (Contribution 23). Collectively, these contributions confirm the great potential of plasmid DNA in oncological gene therapy, where combination therapies are likely to offer the best outcomes.

### 2.6. Broader Plasmid DNA Applications

Broader applications of plasmid DNA technologies were also explored. Gene therapy approaches for monogenic disorders were reviewed, emphasizing challenges such as delivery inefficiencies and strategies to overcome them (Contribution 24). The potential of DNA therapeutics in chronic disease management was discussed, highlighting how these platforms can be tailored for long-term disease modulation (Contribution 25). These contributions illustrate the versatility of plasmid DNA technologies across diverse therapeutic applications.

## 3. Conclusions and Future Directions

The 25 contributions in this Special Issue not only highlight the enormous potential of plasmid-based strategies but also emphasize the need for continued research to overcome the existing challenges that still stand in the way of widespread clinical application of plasmid DNA therapeutics. Even more efficient and targeted delivery systems are needed that improve transfection rates while minimizing unintended responses. The cellular mechanisms underlying nuclear entry and gene expression remain insufficiently understood. Advances in vector design offer promising opportunities to improve expression control, predictability, and therapeutic efficacy. Particularly compelling is the rise in cell-free synthesis platforms producing non-plasmid DNA vectors, which are redefining gene delivery strategies. As the field moves toward industrial-scale production, ensuring clinical-grade DNA with high purity and stability is critical. The regulatory framework will also be crucial to facilitate the translation of these therapies from research to routine clinical practice. Continued multidisciplinary collaboration between molecular biologists, engineers, clinicians, and regulatory experts will be crucial in driving this transformative field forward.

I hope the findings presented in this Special Issue will inspire continued progress in the development of plasmid DNA technologies for a wide range of clinical challenges. Finally, I would like to thank all the authors and reviewers who have contributed to the success of this Special Issue, as well as the journal Pharmaceutics for supporting this endeavor.

